# Efficient Memory-Enhanced Transformer for Long-Document Summarization in Low-Resource Regimes

**DOI:** 10.3390/s23073542

**Published:** 2023-03-28

**Authors:** Gianluca Moro, Luca Ragazzi, Lorenzo Valgimigli, Giacomo Frisoni, Claudio Sartori, Gustavo Marfia

**Affiliations:** 1 Department of Computer Science and Engineering (DISI), University of Bologna, Via dell’Università 50, I-47522 Cesena, Italy; l.ragazzi@unibo.it (L.R.); lorenzo.valgimigli@unibo.it (L.V.); giacomo.frisoni@unibo.it (G.F.); claudio.sartori@unibo.it (C.S.); 2 Department of the Arts (DAR), University of Bologna, Via Barberia 4, I-40123 Bologna, Italy; gustavo.marfia@unibo.it

**Keywords:** abstractive summarization, long document summarization, low-resource summarization, memory-enhanced language models

## Abstract

Long document summarization poses obstacles to current generative transformer-based models because of the broad context to process and understand. Indeed, detecting long-range dependencies is still challenging for today’s state-of-the-art solutions, usually requiring model expansion at the cost of an unsustainable demand for computing and memory capacities. This paper introduces Emma, a novel efficient memory-enhanced transformer-based architecture. By segmenting a lengthy input into multiple text fragments, our model stores and compares the current chunk with previous ones, gaining the capability to read and comprehend the entire context over the whole document with a fixed amount of GPU memory. This method enables the model to deal with theoretically infinitely long documents, using less than 18 and 13 GB of memory for training and inference, respectively. We conducted extensive performance analyses and demonstrate that Emma achieved competitive results on two datasets of different domains while consuming significantly less GPU memory than competitors do, even in low-resource settings.

## 1. Introduction

In the natural language processing (NLP) field, long document summarization (LDS) synthesizes a lengthy input text while retaining relevant information, a critical task to help experts in analyzing massive documents. State-of-the-art (SOTA) solutions are based on transformers [1] and struggle to deal with prolonged documents because of the self-attention mechanism that requires high-memory GPUs to address its quadratic memory growth regarding input size. Most documents, such as contracts and research papers, breach endurable input size limits. This issue has recently opened new research directions towards attention approximations with linear complexity [2,3]. Nevertheless, despite their success, efficient transformers are still GPU-demanding and bound to the input size, e.g., 48 GB for 16 K source tokens [4].

A promising approach to mitigate this issue is exploiting memory-based strategies [5,6]. Specifically, language models are trained to recurrently process a chunk-divided input, writing and reading the past latent knowledge at each step; in this way, the GPU is restricted to working with several length-constrained text fragments instead of elaborating the entire source document at once. Current memory-enhanced models are found on encoder- or decoder-only architectures, preventing their application on sequence-to-sequence tasks such as LDS. Indeed, the most promising research directions for abstractive single- and multi-document summarization currently follow an encoder–decoder paradigm, with lightweight models surpassing or holding up against decoder-only summarizers with hundreds of billions of parameters [7].

**Figure 1:**
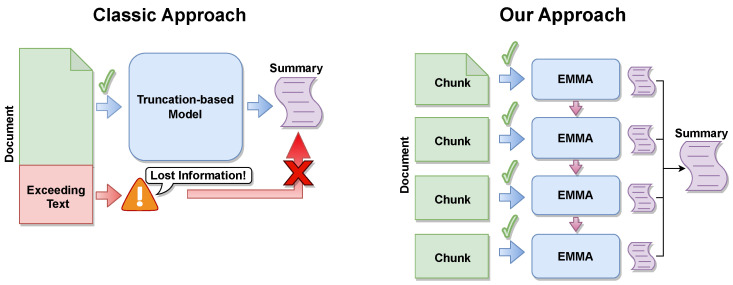
Emma overview, our proposed memory-enhanced approach.

In this work, we present Emma, an efficient memory-enhanced encoder–decoder language model for LDS. Emma reads long inputs chunk by chunk (Figure 1), saving intermediate knowledge and enriching the current context with previous salient information via *cross-memory attention*. We modified the vanilla transformer with new custom memory layers (short- and long-term memory), decoupling the mutual relationship between GPU need and input size.

We experimented on datasets from different domains, showing Emma’s generality and capacity to summarize long inputs with comparable results to strong baselines despite using significantly less GPU memory at training and inference time.

To sum up, our contributions are the following.

- We introduce Emma, a novel memory-enhanced encoder–decoder transformer for LDS.
- We perform extensive analyses showing SOTA’s performance at low GPU cost, on full-resource summarization (i.e., training on all training samples), and few-shot learning.
- The GPU impact of Emma remained fixed regardless of input length.

## 2. Related Work

### 2.1. Transformers

Transformer-based models are the de facto standard in many NLP tasks [8,9]. However, their performance is better as parameters increase, leading to the creation of massive models [7,10]. Despite their success, current works have had problems in dealing with prolonged input sequences because their core layer, namely, self-attention, scales quadratically with input size. For example, the text supplied to Bart must not go beyond 1024 subword tokens, and longer documents have to be cut. Further, most models are pre-trained on sequences of just 512 tokens [10], rendering them unable to handle real-world inputs in downstream tasks. Consequently, meaningful context and details for the summarizers are typically lost. To fill this gap, self-attention has been approximated with linear functions. BigBird [11] and Longformer [4] leverage window-based attention. Nyströmformer [12] uses Nyström-based matrix decomposition. Performer [2] relies on kernel methods. With these notable contributions, large language models can read texts up to 16 K tokens with a GPU of 48 GB memory [4]. Regarding architectures, fine-tuned encoder–decoder models are notoriously dominant compared to zero-shot prompting on large decoder-only language models [13]. Businesses can achieve high summarization quality and versatility with lower costs and more flexibility regarding training and deployment, with networks running locally on private servers and GPUs.

### 2.2. Memory-Based Transformers

The link between memory and neural networks was initially explored with differentiable reading and writing operations in the neural Turing machine [14,15], Differentiable computing networks [16], and gated recurrent units [17]. However, using memory in the transformer is a less investigated research path. TrasformerXL [5] was the first to create a recurrent short-term layer-level memory. In contrast, Compressive Transformer [6] adds long-term memory to the recurrent one. Ernie-Doc [18] improves the memory flow, letting the model deal with infinitely long sequences. *∞*-former [19] leverages a continuous attention framework [20] to create theoretically infinite memory. Importantly, these models are decoder-only and mainly applied to long-input open-generation tasks, thus neglecting LDS. The latest works also focused on top-*k* text-retrieval operations from read-only memories with pre-computed embeddings [21,22]; despite the encouraging performance gain, they rarely support representation updates and have not been tested on document summarization.

### 2.3. Long Document Summarization

SOTA LDS solutions utilize different methods to read long sequences. Hierarchical models [23] iteratively merge paragraph-level dependencies. Segmentation-based approaches [24,25,26] with fusion-in-decoder [27] and marginalized decoding [28] divide the input into meaningful units to produce a summary. Extract-then-abstract procedures [29] pick a subset of relevant sentences from the source to generate the outline, eventually relying on marginalization [30,31]. Lastly, efficient transformers with sparse attention layers [3,4,32] read greater input than quadratic ones do while not fully leveraging the original self-attention mechanism.

## 3. Background

LDS tasks compress a long input text into a coherent short summary. Given the extensive and successful use of the transformer architecture, a document is long if its number of tokens poses processing complications to standard language models. Even if a formal definition does not exist, texts comprising > 1024 tokens are commonly “long”. This threshold is also the maximal input size for well-known quadratic models such as Bart [33] and Pegasus [34].

The problem of LDS can be formalized with an input document X and its target summary Y. Since a classical transformer needs to rely on input truncation, memory can help in preserving salient information. Intuitively, we can split a long input into chunks $\left\{c_{1},c_{2},\cdots ,c_{n}\right\}$ and give them one by one to a model that could (i) read each chunk, (ii) save the relevant information in the memory and reuse it for subsequent chunks, and (iii) generate a summary for each chunk. Eventually, the final summary is obtained by concatenating chunk-level summaries.

Unfortunately, existing memory-based transformers are limited to (X,Y) tasks with a target for each input text. This setting is a substantial limitation and the main reason why memory-based transformers have not yet been applied to LDS where there is a single target even after segmentation.

## 4. Method

Emma is a novel efficient memory-augmented transformer for LDS. Our model relies on a text segmentation algorithm and memory layers to recurrently read the provided input, chunk after chunk; at each step, it stores the relevant information and compares it with previous information. Emma can handle infinitely long documents with a fixed amount of GPU memory.

### 4.1. Text Segmentation

Let $X=\{x_{1},\cdots ,x_{x}\}$ and $Y=\{y_{1},\cdots ,y_{y}\}$ be the long input document and related target summary, respectively, where each $x_{i}\in X$ and $y_{i}\in Y$ is a sentence. We segmented X into non-overlapping chunks C of max $L_{c}$ tokens with a sentence-level segmentation algorithm (Algorithm 1). We started with an empty chunk *c* and iteratively added sentences until $L_{c}$. After constructing the chunks, we paired each target summary sentence with the chunk that maximized the ROUGE-1 precision metric [26], deriving small source-target pairs. Consequently, we turn the problem from $\left\{\left(c_{1},c_{2},\cdots ,c_{n}\right),Y\right\}$ to $\left\{\left(c_{1},t_{1}\right),\left(c_{2},t_{2}\right),\cdots ,\left(c_{n},t_{n}\right)\right\}$, where $c_{1}\circ c_{2}\circ \cdots \circ c_{n}=X$ and $t_{1}\circ t_{2}\circ \cdots \circ t_{n}=Y$, with ∘ denoting string concatenation.
**Algorithm 1** Text Segmentation**Input**: $X=\{x_{1},\cdots ,x_{x}\}$ **Parameters**: $L_{c}$ **Output**: C▹ Input sentences
▹ Number of tokens per chunk ▹ Set of chunks 1:C←∅ 2:c←∅ 3:**for** $x_{i}\in X$**do** 4:    $l\leftarrow len\left(c\right)+len\left(x_{i}\right)$ 5:    **if** $l<L_{c}$ **then** 6:        $c\leftarrow c\circ x_{i}$ 7:    **else** 8:        C←C+c 9:        c←∅10:    **end if**11:**end for**12:**if** len(c)≠∅ **then**13:    C←C+c14:**end if**15:**return** C

### 4.2. Model Architecture

**Figure 2:**
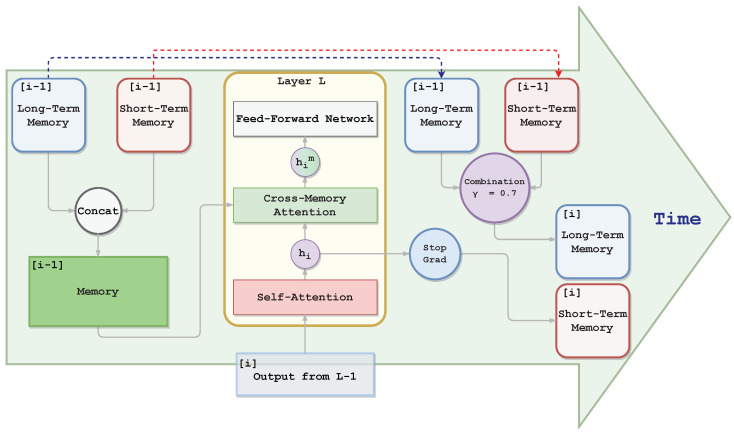
Graphical representation of our proposed memory-enhanced layer. (left to right) Long- and short-term memories are (i) concatenated, (ii) fused with input hidden representation via cross-memory attention, and (iii) the input hidden representation becomes the new short-term memory while the long-term memory is updated with information from the previous short-term one. To simplify the figure, we do not depict the residual connections.

We enhanced the transformer-based model Bart [33] with a recurrent layer-level memory where the model stores past information. Specifically, we allowed for the model to compare current chunk $c_{i}$ with information related to previous ones $\left\{c_{1},\cdots ,c_{i-1}\right\}$. The original layers of the Bart encoder are composed of self-attention and feed-forward blocks with residual connections. As shown in Figure 2, we added a layer-level memory and a second attention block, termed *cross-memory attention*, to perform reading and writing operations. The memory is a single matrix M.

#### 4.2.1. Cross-Memory Attention

We added cross-memory attention after a residual connection that follows the self-attention of the classical Bart encoder layer. At the *i*-th step, this module enables the model to juxtapose the hidden states $h_{i}$ of chunk $c_{i}$ with $\left(h_{1},\cdots ,h_{i-1}\right)$ via cross-attention. Around this layer, we added a residual connection to let the model learn how much to use the memory. Formally, hidden state $h_{i}^{m}$ is acquired with the following formula:

(1) $$h_{i}^{m}=N\left(h_{i}+C\left(h_{i},M_{i-1}\right)\right),$$

where N is a normalization layer, C is the cross-memory attention layer, $M_{i-1}$ is the memory, and $h_{i}$ is the hidden state after the self-attention.

#### 4.2.2. Memory Writing

We equipped each layer with a memory to store helpful information for the next step, overriding the previous memory. After performing cross-memory attention for the *i*-th chunk and generating $h_{i}^{m}$, $h_{i}$ is given to the memory module. In detail, $h_{i}$ passes through a stop gradient function, $SG(h_{i})$, and becomes the new memory matrix:

(2) $$M_{i}=SG\left(h_{i}\right).$$

By stopping the gradient, the GPU memory used at training does not increase with the number of chunks, allowing for the model to work with a theoretically infinitely size document.

#### 4.2.3. Long-Term Memory

With the memory overridden at each step, we may lose long-term details. For this reason, we improved the architecture by adding a long-term memory. In particular, we moved $M_{i-1}$ into a different matrix $M_{i}^{l}$, which we call the long-term memory matrix, before overriding it with the new hidden state $h_{i}$. Memory $M_{i-1}$ was compressed and combined with the long-term memory matrix $M_{i-1}^{l}$ as follows:

(3) $$M_{i}^{l}=\left(1-\gamma \right)\cdot M_{i-1}^{l}+\gamma \cdot M_{i-1},$$

where γ is a compress ratio empirically set to 0.7. The final memory $M_{i}^{c}$ used for the cross-memory attention was obtained by concatenating the short- and long-term memories:

(4) $$M_{i}^{c}=C\left(M_{i-1},M_{i-1}^{l}\right),$$

where C is the concatenation function.

### 4.3. Training and Inference

Emma takes as input the chunk–target pairs and was trained to generate the next output token for each target by minimizing the negative log-likelihood:

(5) $$L=-\frac{1}{\left|t\right|}\sum_{i=1}^{\left|t\right|}\log p\left(y_{i}|y_{1:i-1},c\right),$$

where *c* is the input chunk, and $y_{1:t}$ are the tokens from position 1 to *t* of its target *t*. For the training process, we took only the chunk–target pairs $\left(c_{i},t_{i}\right)$, such that $t_{i}\neq \emptyset$. Instead, at inference time, we considered all the chunks and concatenated the chunk-level summaries to establish the final prediction.

### 4.4. Space Complexity

Our model, Emma, has quadratic space complexity regarding the length of the input chunks. Given a predefined max chunk size $L_{c}$, a document with size $L_{D}$ is split at most into $\left\lceil \frac{L_{D}}{L_{c}}\right\rceil$ chunks. Thanks to our solution, the chunks are individually processed and synthesized, and their summaries are concatenated to produce the final output (Figure 1). Hence, the space complexity to summarize the entire input document is $O(L_{c}^{2})$; since it relies on the model’s encoder self-attention for a single chunk, it remains fixed regardless of the document length. As our model was built upon Bart, the encoder self-attention had quadratic complexity in the chunk size.

## 5. Experiments

### 5.1. Evaluation Datasets and Training Settings

We tested Emma under (i) full training and (ii) few-shot learning scenarios by utilizing datasets containing long documents on different specific domains. In (i), we took GovReport [3] and PubMed [35] as the evaluation benchmarks. GovReport collects reports from government research agencies, while PubMed comprises biomedical research articles. In (ii), we worked with BillSum [36], which consists of U.S. congressional bills. Statistics of the datasets are reported in Table 1. To reduce the training time and energy consumption, we used a maximum of 20 K training instances for each dataset. For GovReport, we used the default training and test splits: the training set comprised 17,517 instances, and the test set contained 973 examples. For PubMed, we used the first 20,000 samples of the training set and the full test set of 6658 instances. For BillSum, following prior works [34,37], we utilized the first 10 and 100 training instances (the same sampling strategy as that for validation).

We adopted the ROUGE-1/2/L standard [38] as the automatic LDS metric. Inspired by [39], we also computed $R=\mathrm{avg}(r_{1},r_{2},r_{L})\;/\;1+\sigma_{r}^{2}$, where $\sigma_{r}^{2}$ is the ROUGE F1 score variance. In this way, we derived an aggregated judgment that, in the case of equal $r_{1/2/L}$ averages, penalizes generations with heterogeneous results across dimensions. To contain the variance effect that was only designed to slightly refine average values, we considered $r_{1/2/L}\in \left[0,1\right]$ and R∈[0,1] (the higher, the better). Lastly, we performed qualitative analysis to complement the notorious lexical superficiality of ROUGE [40].

### 5.2. Baselines

- *Full training*. To understand the contribution of our new memory, we examined Bart [33], the skeleton model that we had extended. Then, we contemplated SOTA models on Bart that do not perform any further pre-training, like ours. We chose Led [4] and Hepos [3], which leverage various efficient attention mechanisms and are capable of reading the entire long input. In particular, in Hepos, we considered locality-sensitive hashing (lsh) and sinkhorn. We lastly evaluated our model against Summ${}^{N}$ [41], a segmentation-based solution.
- *Few-shot learning*. We compared it with well-known low-resource abstractive summarizers. Pegasus [34] is a transformer-based model with a summarization-specific pre-training objective that allows for fast adaption through a few labeled samples. Mtl-Abs [37] combines transfer learning and meta-learning from multiple corpora by using adapter modules as bridges. To judge the contribution of document segmentation versus memory, we contrasted Emma with Se3 [26], a semantic self-segmentation approach for LDS under low-resource regimes with proven strength in data scarcity conditions. Similarly to our model, Se3 avoids truncation by creating highly correlated source–target chunk-level pairs with lengths modulated to fit into the GPU memory. Despite empowering the chunk definition process with deep metric learning following information retrieval techniques [42,43,44,45], Se3 represents a general pre-processing technique for any transformer where chunks are individually summarized and then concatenated (no memory extension or architectural changes). To ensure fairness, we refer to Se3+Bart.

### 5.3. Experimental Settings

We trained Emma for 10 epochs in two versions, the base (192 M trainable parameters) and large (508 M trainable parameters). We report the results of the best-performing checkpoint on the validation set. We used the AdamW optimizer with $\beta_{1}=0.9$ and $\beta_{2}=0.99$, and set the dropout to 10%. The learning rate was $3\times 10^{-5}$, the batch size was 1, and the seed was fixed to 42 for reproducibility. At inference time, we set the beam width to 5 for all experiments and prevented the repetition of n-grams of size 5. We used a summary length between 400 and 1000 for GovReport, and 100 and 700 for PubMed with the repetition penalty set to 1. We conducted the work on a workstation using a single GPU RTX 3090 with 24 GB dedicated graphics memory, 64 GB RAM, and an AMD EPYC 7443 24-core processor. The operative system was Ubuntu 20.04.3 LTS; the development environment was a docker container with an official Hugging Face image (huggingface/transformers-pytorch-latest-gpu, accessed on 13 March 2023). We implemented the code using Python 3.8, PyTorch to handle gradient optimization, and Hugging Face for the neural models (https://huggingface.co/models, accessed on 13 March 2023) and datasets (https://huggingface.co/datasets, accessed on 13 March 2023).

### 5.4. Performance Evaluation

We extensively measured Emma’s performance quantitatively and qualitatively. All ROUGE scores detailed in this section are expressed as percentages.

#### 5.4.1. Full-Training Results

Table 2 reports the LDS results under full-training settings. Compared to traditional SOTA encoder–decoder summarizers without memory, Emma achieved competitive or higher ROUGE F1 scores, with significant improvements in hardware requirements (see Section 5.5). The outcomes show that Emma captures salient information if either equally distributed in the long input (GovReport) or accumulated in the first partitions of documents (PubMed).

#### 5.4.2. Few-Shot Learning

By supervising our model on limited data, we analyze how quickly Emma leverages the inner pre-trained model. Results in Table 3 indicate that Emma outperforms previous summarizers, revealing its learning skills in low-resource. Higher ROUGE scores over Se3 corroborate the memory value more than segmentation only does.

#### 5.4.3. Ablation Studies

To assess the importance of our architecture’s main components, we performed a set of ablation studies (Table 4 and Table 5), using the GovReport training settings with 1000 samples for 3 epochs. In particular, we investigated the following design choices.

- *w/Backprop*: We attempted not to stop the backpropagation within the current chunk but allowed it to go back in time to previous steps. Results show a performance drop, probably due to the increased learning complexity. This approach is unexplored in memory-enhanced transformers and deserves greater research attention.
- *w/Long-term memory*: we removed the long-term memory module. Results worsened, ascertaining the contribution of this component to the final summary quality.
- *Memory layers*: We performed a series of experiments to determine which layers turned the memory on. The last two were the best ones, aligned with Rae and Razavi [46], where the authors claimed that TransformerXL operated better with memory only on layers in the second half of the encoder.

### 5.5. Analysis of the GPU Impact

#### 5.5.1. GPU Memory Usage

**Figure 3:**
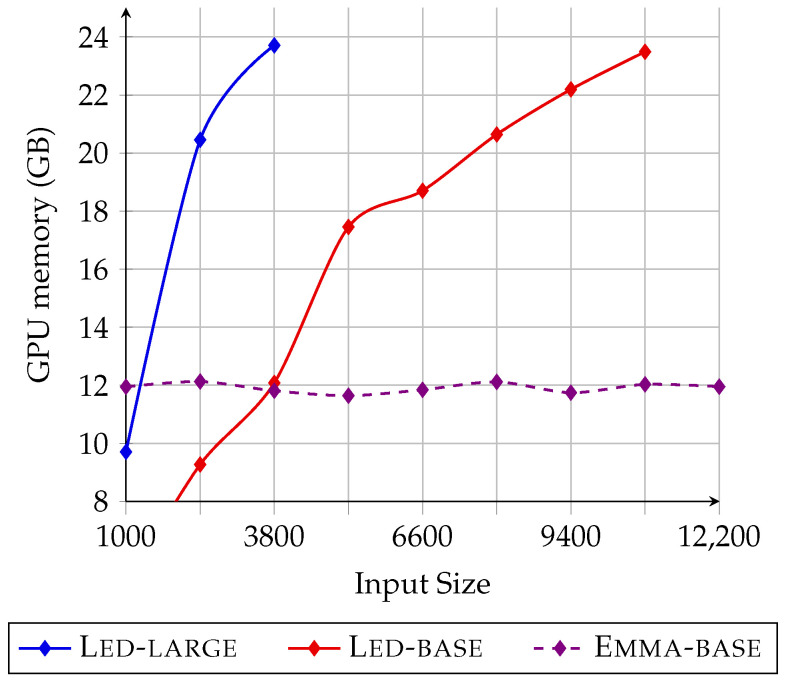
GPU memory occupation at training time by varying input size (batch_size=1).

One of the main benefits of adopting memory components into language models is that the GPU memory consumption rate remains fixed regardless of the input document length. SOTA solutions with efficient attention mechanisms, such as Led and Hepos, have a maximal limit on the number of tokens that they can read simultaneously. Therefore, applying such models to domains characterized by extremely long sources (e.g., books, meeting dialogues, trials) is hard if not impossible. Memory can precisely mitigate this problem: at inference time, theoretical GPU usage depends only on the dimension of the model. This property held for our solution, even during training, thanks to interrupting the backpropagation through chunks. Figure 3 qualitatively exhibits the training time of GPU utilization for 10 artificially crafted documents ordered by length. We compared our model with the best-performing linear attention transformers, namely, Led-base and Led-large (retrained by us). Emma’s GPU need was stable for all documents despite the increase in source tokens.

In linear-attention-based solutions such as Led, memory usage scales linearly regarding input length. However, these models still suffer from serious scalability issues that preclude their application. For example, according to their original papers [3,4], both Hepos (batch size 2) and Led (batch size 1) require 48 GB of GPU memory to fit and train the models for processing 16 K input tokens. Moreover, their functions for approximating quadratic self-attention perform slightly worse with short inputs. Similarly, Dyle uses 48 GB with batch size 8. Its memory usage depends on the number of top-*K* snippets to select from the input source. In our 24 GB hardware configuration, only K=10 was manageable corresponding to F1 ROUGE scores equal to 54.98/24.10/51.25 [30], which were significantly worse than those of Emma in the same settings.

Our solution achieves comparable results on GovReport using less than 24 GB of GPU memory. Similar to Dyle, our GPU memory consumption did not scale with the document length, but the minimal amount required was significantly lower.

#### 5.5.2. Chunk Size Analysis

**Figure 4:**
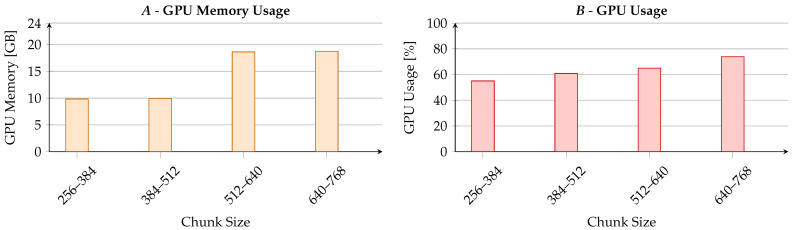
Graphs on how the chunk size hyperparameter of Emma impacts the GPU. (**A**) GPU memory usage (0–24 GB); (**B**) GPU computational power usage (0–100%).

We split the input document into chunks; the memory used at inference time only depended on the one needed to process a single chunk. Table 6 shows how the performance changed by varying the chunk length. Since we segmented the input document, the memory used at the inference time only depended on the one needed to process a single chunk. Table 6 depicts how summarization effectiveness changed by varying the chunk size bounds. ROUGE scores slightly worsened by decreasing the number of tokens per chunk, but our model powerfully maintained a good trade-off between chunk size and summary quality. Leveraging past information thanks to memory is vital for generating high-quality summaries, especially when decreasing the chunk size (i.e., increasing the number of chunks). In a nutshell, Emma achieves highly competitive summarization performance even with reduced chunk sizes, which implies downsized GPU memory demand. Figure 4 shows the impact of the chunk size from an efficiency perspective, measuring the GPU usage and memory occupation. A significant GPU memory drop appeared with a chunk size between 384 and 512 tokens. Further, the GPU usage scaled linearly with the chunk size. Chunks between 384 and 512 tokens had the best trade-offs. These outcomes show that memory can be central in low-resource models, uncoupling the GPU impact and the input length.

### 5.6. Human Evaluation

**Figure 5:**
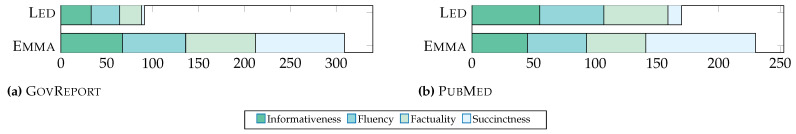
Distribution of votes per quality dimension (cumulative best-selection percentages).

**Figure A1:**
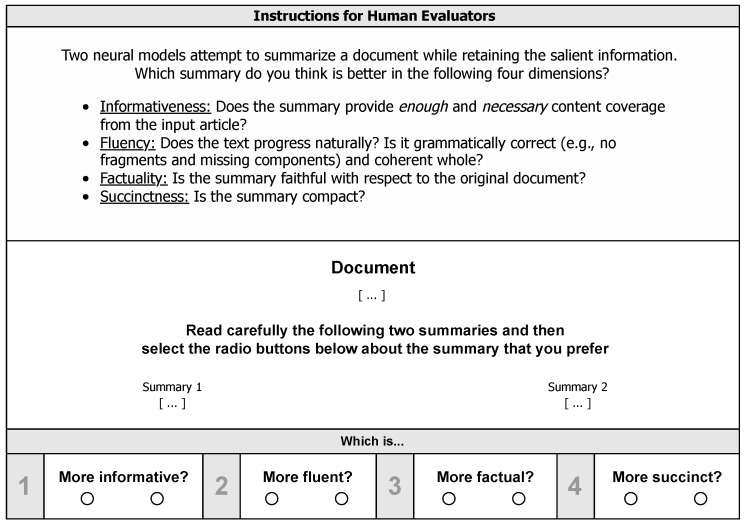
Human assessment interface.

We conducted a comprehensive human evaluation study to better gauge the quality of the summaries produced by Emma regarding Led (the main full-training competitor according to Table 2). We randomly selected 50 document–summary pairs from the test sets of GovReport and PubMed (25 from each source). We asked three evaluators who were proficient in English with legal and medical competencies to select their most and least preferred predictions according to informativeness, fluency, factuality, and succinctness, i.e., best–worst scaling [47,48]. We randomized the order of summaries within pairs to guard the rating against being gamed. Our setup with human instructions is illustrated in Figure A1. The annotation process took approximately 6 h per judge, 18 h in total. The average Kendall coefficient among all evaluators’ inter-rater agreement was 0.60. All evaluation files were publicly released for transparency and reproducibility: https://github.com/disi-unibo-nlp/emma (accessed on 13 March 2023). Results are outlined in Table 7, showing the overall percentage of times that a particular system was the most preferred summary source. Additionally, we plot the distribution of dimension-specific votes in Figure 5. Across both quality dimensions and datasets, we observed a clear preference for Emma. Led tended to be less abstractive and to have more extended outputs, often cut before reaching the end-of-sentence token, focused on the first part of the document. Instead, Emma was much more concise, going straight to the point and covering all the relevant content mentioned in the document with high frequency and factuality. The overall advantage of our solution is strongly accentuated as the length of the target summary increased (GovReport summaries were, on average, 2.58× longer than those of PubMed).

## 6. Conclusions

Although augmenting transformers with memory is receiving less attention and effort than efficient transformers, it can play a pivotal role in low-resource settings and domains with extremely long documents. In this work, we presented Emma, the first memory-enhanced encoder–decoder transformer for long-document summarization. The proposed architecture leverages two fundamental elements: (i) a segmentation algorithm for splitting the input document into chunks and pairing them with the most related parts of the target summary, and (ii) a recursive memory module capable of storing information from past chunks. We tested our solution with multiple datasets of different domains, obtaining competitive results with state-of-the-art models under full-training conditions and outperforming prior works in few-shot learning. Exceptionally, depending only on the chunk size, the GPU need remained constant regardless of the whole document length. Compared to segmentation-only techniques, our memory component boosted summarization quality, avoiding treating each chunk independently, and better exploiting their semantic linkage. We also verified that the chunk size could be kept low without significant drops in summarization results, enabling SOTA performance on limited hardware. In-depth ablation studies support our architectural design choices. This study promotes novel research toward efficient memory-enhanced language models.

## 7. Limitations and Future Directions

Our document segmentation algorithm requires the length of the golden summary to not be too short; otherwise, paired targets are composed of a few tokens. According to our empirical tests in the ablation studies, enabling backpropagation through chunks led to worse results. Deeper investigations are needed to solve this issue.

Future works should explore memory writing/reading operations with structured information extracted from text, comparing unsupervised techniques for document metadata acquisition (e.g., classes [49,50] and entity relationships [51,52]) with advanced semantic parsing solutions such as event extraction [53,54] and abstract meaning representation, which was recently used for knowledge injection into deep neural networks [55,56]. The community should envisage novel graph representation learning methods [57,58,59,60] to densely represent multi-relational structured data following a Linked Open Data vision centered on the integration of several source knowledge graphs or relational databases via automatic entity matching [61]. Taking inspiration from biology [62,63] and communication networks [64,65,66,67], we underline the importance of managing dynamic scenarios, tracking knowledge refinements among sentences, and propagating information, which is pivotal when processing lengthy inputs. Segmentation strategies and memory-enhanced encoder–decoder transformers could be inspected in other downstream tasks with long documents and cross-dependencies among chunks, such as claim verification with evidence retrieval [68,69].

## Figures and Tables

**Table 1:** Statistics of the LDS datasets used as evaluation benchmarks. (top) Full training; (bottom) few-shot learning.

Dataset	Samples	Source	Target
		*#avg Words*	*#avg Words*
GovReport	19,466	9409.4	553.4
PubMed	133,215	3224.4	214.4
BillSum	23,455	1813	207.7

**Table 2:** Full-training ROUGE F1 scores on GovReport and PubMed. Baseline results are from the original papers. Bold and underline denote the best and second-best scores.

	GovReport		PubMed		Average R
Model	R1/R2/RL	R	R1/R2/RL	R	
**Baselines**					
Bart [33]	52.83/20.50/50.14	40.29	45.36/18.74/40.26	34.33	37.31
Hepos-lsh [3]	55.00/21.13/51.67	41.63	**48.12**/**21.06**/42.72	**36.80**	39.99
Hepos-sinkhorn [3]	56.86/22.62/53.82	43.39	47.96/20.78/42.53	36.59	39.99
Led [4]	**59.42**/**26.53**/**56.63**	**46.50**	47.00/20.20/42.90	36.20	**41.35**
Summ${}^{N}$ [41]	56.77/23.25/53.90	43.64	–	–	–
**Ours**					
Emma-base	58.78/24.30/55.29	45.04	44.31/17.35/40.91	33.70	39.37
Emma-large	59.39/25.27/55.90	45.77	46.70/19.51/**43.42**	36.01	40.89

**Table 3:** Few-shot learning ROUGE F1 scores on the BillSum dataset using 10 and 100 training instances. Baseline results are from the original papers. Bold and underline denote the best and second-best scores.

	BillSum (10)		BillSum (100)		Average R
Model	R1/R2/RL	R	R1/R2/RL	R	
**Baselines**					
Pegasus [34]	40.48/18.49/27.27	28.52	44.78/26.40/**34.40**	34.99	31.76
Mtl-Abs [37]	41.22/18.61/26.33	28.47	45.29/22.74/29.56	32.24	30.36
Se3 [26]	46.58/22.03/28.23	31.93	49.88/26.84/33.33	36.34	34.14
**Ours**					
Emma-base	**46.77**/**22.95**/**28.81**	**32.51**	**50.78**/**28.58**/34.27	**37.55**	**35.03**

**Table 4:** Ablation studies to validate the components of the solution. The best results are in bold.

	GovReport		
Model	R1	R2	RL
Full	**59.99**	**23.96**	**56.35**
w/Backprop	41.44	12.66	39.98
w/o Long-term memory	58.83	22.61	55.03

**Table 5:** Ablation study to assess which layer turns on the memory. The best results are in bold.

	GovReport		
Memory-Layer	R1	R2	RL
All	58.71	23.18	55.73
Last three	59.22	**24.10**	55.96
Last two	**59.99**	23.96	**56.35**
Last one	58.76	22.91	55.51

**Table 6:** Results of over 100 documents of the test set from GovReport while changing chunk size.

Chunk Size	R1	R2	RL
256–384	58.43	23.44	54.31
384–512	60.92	25.21	56.93
512–640	61.65	26.50	58.12
640–768	61.46	26.15	58.21

**Table 7:** Percentage of times that a summarizer was selected as the best from all evaluators. Annotators preferred Emma outputs over Led for approximately 70% of the sampled document–summary pairs. The best results are on a green background.

Model	GovReport	PubMed	Overall
Led	22.67	42.33	32.50
Emma	77.33	57.67	67.50

## Data Availability

All pre-trained models and corpora used in this work are publicly available (see Appendix A).

## Abbreviations

The following abbreviations are used in this manuscript:

LDS	Long document summarization
LSH	Locality-sensitive hashing

## Appendix A. References to Models and Datasets

Table A1 enumerates all the trained models and datasets used in this study, linking to specific HuggingFace versions.

**Table A1:** List of the models and datasets used in this study. All the links have been last accessed on 13 March 2023.

Model	URL
Led-base	https://huggingface.co/allenai/led-base-16384
Led-large	https://huggingface.co/allenai/led-large-16384
Bart-base	https://huggingface.co/facebook/bart-base
Bart-large	https://huggingface.co/facebook/bart-large
**Dataset**	**URL**
GovReport	https://huggingface.co/datasets/ccdv/govreport-summarization
PubMed	https://huggingface.co/datasets/ccdv/pubmed-summarization
BillSum	https://huggingface.co/datasets/billsum

## Appendix B. Human Evaluation Insights

The interface with human evaluation instructions is sketched in Figure A1.
